# Expression of Concern: Vitamin D sufficiency, a serum 25-hydroxyvitamin D at least 30 ng/mL reduced risk for adverse clinical outcomes in patients with COVID-19 infection

**DOI:** 10.1371/journal.pone.0240965

**Published:** 2020-10-14

**Authors:** The PLOS ONE Editors

After this article [1] was published, concerns were raised about the validity of results and conclusions reported in the article and about undisclosed competing interests.

Specifically,

- Concerns were raised about the reported study’s sample size and whether it was adequate to address the aims of the study.
- Questions were raised about whether the statistical analyses and results were sufficiently robust to support the article’s conclusions, and about how potential confounds were addressed in the data analyses. As one aspect of this, it was raised that vitamin D levels may be indicative of co-morbidities that may themselves impact COVID outcomes.
- There are statements in the article, including in its title and conclusions, that suggest a causal relationship between vitamin D levels and the clinical outcome of COVID-19 infections which is not supported by the data.
- Of the participants in the study, only 31.06% had RT-PCR tests confirming a COVID-19 diagnosis. As such, the COVID-19 status of other participants is in question, although in the article all are reported as patients with COVID-19. This is not taken into consideration in the subgroup analyses as reported in the article, and calls into question the overall interpretation of the results.
- The article’s Competing Interests statement says, “The authors have declared that no competing interests exist.” However, publicly available information indicates that corresponding author MFH may have potential competing interests that include non-financial interests based on his vitamin D research and other activities focused on vitamin D; contributions to an app that tracks vitamin D; and interests that include consultancies, funding support, and authorship of books related to vitamin D usage.

The authors commented that the article did not claim a causal role of vitamin D on clinical outcomes, and that the limitations of the study were clearly described in the Discussion which said, “we cannot explain the cause and effect relationship of vitamin D sufficiency and the reduced risk of severity from a COVID-19 infection.” [1] They also stated that COVID-19 diagnoses were made by infectious disease specialists per WHO interim guidance and recommendations of the Iranian National Committee of COVID-19 [2].

*PLOS ONE* is currently reassessing the article and following up on the above issues in accordance with COPE guidance and journal policies. Meanwhile, the *PLOS ONE* Editors issue this Expression of Concern.

## References

[1] Maghbooli Z, Sahraian MA, Ebrahimi M, Pazoki M, Kafan S, Tabriz HM, et al (2020) Vitamin D sufficiency, a serum 25-hydroxyvitamin D at least 30 ng/mL reduced risk for adverse clinical outcomes in patients with COVID-19 infection. PLoS ONE 15(9): e0239799 10.1371/journal.pone.0239799 32976513 PMC7518605

[2] Talebpour M, Hadadi A, Oraii A, Ashraf H. Rationale and Design of a Registry in a Referral and Educational Medical Center in Tehran, Iran: Sina Hospital Covid-19 Registry (SHCo-19R). Adv J Emerg Med. 4(2s):e53.

